# 927. Successful Implementation of an Inpatient Fluoroquinolone (FQ) Pre-Authorization (PA) Program at a Tertiary Care Academic Center and Associated Changes in Antibiotic Use, Antibiogram Susceptibility, and Provider Behavioral Changes

**DOI:** 10.1093/ofid/ofac492.772

**Published:** 2022-12-15

**Authors:** Lindsay Taylor, Jessica S Tischendorf, Lucas Schulz, Nasia Safdar, Alexander Lepak

**Affiliations:** University of Wisconsin School of Medicine and Public Health, Madison, Wisconsin; University of Wisconsin School of Medicine and Public Health, Madison, Wisconsin; UW Health, Madison, Wisconsin; University of Wisconsin School of Medicine and Public Health, Madison, Wisconsin; University of Wisconsin School of Medicine and Public Health, Madison, Wisconsin

## Abstract

**Background:**

In 2017, our adult tertiary care academic hospital in the Midwest implemented an inpatient fluoroquinolone (FQ) prior authorization (PA) policy using the electronic health record (EHR) as a stewardship tool. We examined the changes in antibiotic use, the antibiogram, and antibiotic use at an affiliated hospital without PA but staffed by physicians from the PA site.

**Methods:**

This quasi-experimental study used a pre-post implementation design to evaluate a PA policy at our University Hospital requiring approval of all inpatient FQ use from the antimicrobial stewardship physician or infectious disease consult teams. Time periods consisted of 2.5 year pre-implementation, 9-month wash-in (pilot on 2 wards), and 5-year post-implementation periods. Monthly antibiotic use in days of therapy per 1000 patient days (DOT/KPD) and antibiogram data, limited to first inpatient culture per patient per week from any anatomic site, were collected. Monthly FQ use from an affiliated hospital without the restriction policy staffed by physicians from the University Hospital was also collected. Changes in antibiotic use were examined using t-test, or Mann-Whitney Rank Sum, and antibiotic susceptibility rates were compared using z-test.

**Results:**

Following implementation of PA, FQ use decreased by 76% (-53.2 DOT/KPD, p< 0.001) (Fig. 1). FQ use also declined from 26.4 DOT/KPD to 7.7 DOT/KPD (p< 0.001) at the affiliated hospital without PA but staffed by physicians from University Hospital. Changes in gram-negative agents use are shown in Table 1, with greatest increase noted for ceftriaxone (∼50%). The ciprofloxacin inpatient antibiogram improved significantly (Table 2); whereas, a slight decline in susceptibility was noted for ceftriaxone and cefepime.

**Figure 1.**
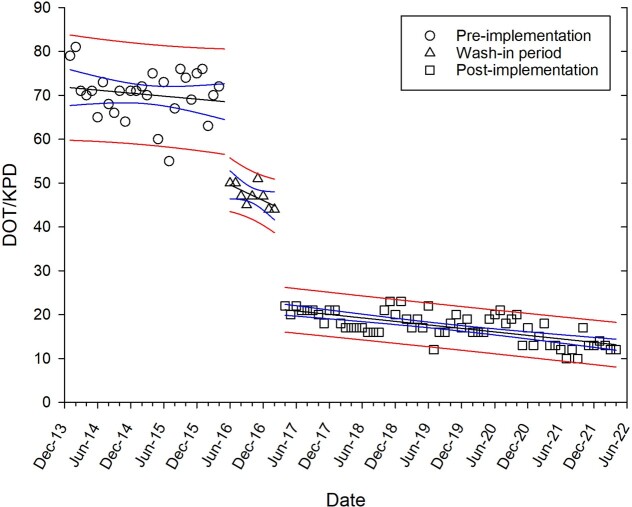
Fluoroquinolone use across study periods.

Wash-in period included a pilot fluoroquinolone prior authorization in 2 wards.

Abbreviations: Days of Therapy (DOT); patient days (PD)

**Table 1.**
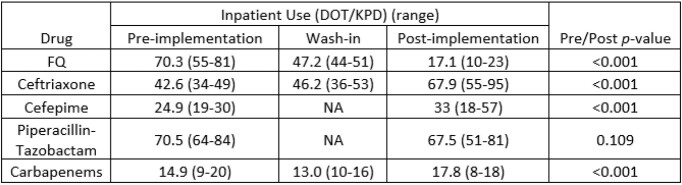
Mean antibiotic days of therapy during each study period.

Successive shortages for cefepime and piperacillin-tazobactam during the wash-in period, as indicated by NA.

**Table 2.**
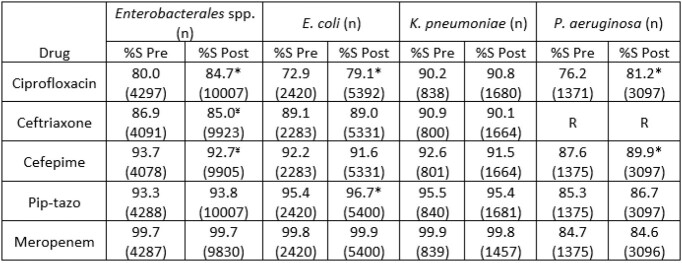
Proportion of susceptible isolates pre- and post-implementation of Fluoroquinolone prior-authorization.

**Conclusion:**

FQ PA leads to significant and sustained decline in FQ use, indicating that suitable alternative choices exist for most hospitalized patients. Decreased FQ use was associated with increased use of cephalosporins, mainly ceftriaxone. As might be expected, changes in use were associated with subsequent changes in the antibiogram. This intervention was associated with a significant decline in FQ use at a site without the restriction policy and the EHR modifications, suggesting successful diffusion of educational and behavioral changes.

**Disclosures:**

**Lindsay Taylor, MD**, Merck: Grant/Research Support **Jessica S. Tischendorf, MD, MS**, Merck: Grant/Research Support.

**Table 2.** Proportion of susceptible isolates pre- and post-implementation of Fluoroquinolone prior-authorization.

Pip-tazo, piperacillin-tazobactam; %S, the percent susceptible for Pre- or Post-implementation periods; * indicates significant improvement and ¥ significant decline (p<0.05) pre vs post; R indicates intrinsic resistance.

